# Cross‐Sectional Study on the Relationship of Some Prognostic Factors of Endodontic Treatment With the Presence and Size of Periapical Lesions in Endodontically Treated Mandibular Anterior and Premolar Teeth Using Cone‐Beam Computed Tomography

**DOI:** 10.1155/ijod/9937176

**Published:** 2026-02-06

**Authors:** Nazanin Zargar, Ali Akbar Ahmadi, Ayeh Etemadi, Yaser Safi, Seyedeh Sareh Hendi, Alireza Akbarzadeh Baghban

**Affiliations:** ^1^ Department of Endodontics, School of Dentistry, Shahid Beheshti University of Medical Sciences, Tehran, Iran, sbmu.ac.ir; ^2^ Department of Oral and Maxillofacial Radiology, School of Dentistry, Shahid Beheshti University of Medical Sciences, Tehran, Iran, sbmu.ac.ir; ^3^ Proteomics Research Center, Department of Biostatistics, School of Allied Medical Sciences, Shahid Beheshti University of Medical Sciences, Tehran, Iran, sbmu.ac.ir

**Keywords:** cone-beam computed tomography, cross-sectional study, lesion volume, periapical periodontitis, prognostic factor, root canal therapy

## Abstract

**Objectives:**

This study evaluated the association between key prognostic factors in nonsurgical endodontic treatment and the presence and size of periapical lesions (PALs) in mandibular anterior and premolar teeth using cone‐beam computed tomography (CBCT).

**Materials and Methods:**

A cross‐sectional analysis was conducted on 63 CBCT scans covering 105 mandibular anterior and premolar teeth. Variables examined included missed canals, underfilled or overfilled canals, procedural errors (e.g., perforations and fractured instruments), intracanal posts (cast or prefabricated), and restoration type. The presence and volume of PALs were assessed, and associations were evaluated using a general linear model (GLM) and multiple logistic regression (*α* = 0.05).

**Results:**

Among 63 patients (27 males and 36 females), PALs were observed in 43 teeth (41%) with an average lesion volume of 7.90 mm^3^. No significant association was found between PAL occurrence and age, gender, tooth type, restoration type, or intracanal posts (*p*  > 0.05). However, missed canals (*p* = 0.042), underfilled (*p*  < 0.001), overfilled canals (*p*  < 0.001), perforations (*p* = 0.020), and fractured instruments (*p* = 0.033) were significantly associated with PAL presence. Lesion size correlated significantly with gender (*p* = 0.047), overfilling (*p* = 0.013), fractured instruments (*p*  < 0.001), and apical perforations (*p* = 0.009).

**Conclusions:**

The findings underscore the clinical relevance of missed canals, over‐ and underfilling, and procedural mishaps in the development and size of PALs. Patient gender also appeared to influence lesion size. These results highlight the need for precision in endodontic procedures to improve periapical healing.

## 1. Introduction

Apical periodontitis (AP) is a chronic inflammatory response triggered by microorganisms present in infected root canal systems [1, 2]. The primary goal of root canal treatment is to eliminate or minimize the microbial load in the root canal system through chemomechanical debridement and subsequent root canal sealing [3]. Contamination of the root canal system may occur in teeth with or without previous root canal treatment [4]. Despite the advancements in root canal treatment techniques, an increase has been reported in the prevalence of AP in the literature [5]. According to a meta‐analysis by Jakovljevic et al. [6], the prevalence of AP in the adult general population increased from 5.4% in 2012 to 6.3% in 2020, while its prevalence in endodontically treated teeth rose from 35.9% to 41.3%.

Factors associated with the success of root canal treatment and prevalence of periapical diseases include poor density of root filling, over‐extension or under‐extension of root canal filling, intraoperative procedural errors, poor‐quality coronal restoration, and missed root canals [7, 8]. The size of periapical lesion (PAL) is a strong independent predictor of endodontic treatment outcome, influencing periapical tissue healing following nonsurgical endodontic treatment [9–11]. Previous studies have shown that smaller lesions (<5 mm) are associated with a higher healing rate, with success rates ranging from 80% to 86%; whereas, larger lesions (> 5 mm) exhibit lower success rates, approximately 65%–70%, following nonsurgical endodontic treatment [12–15]. Larger lesions are more susceptible to insufficient curettage, and the remaining tissue can serve as a source of infection, decelerating the healing process. Lesions with a mean volume larger than 60 mm^3^ have shown a significantly lower success rate in periapical surgery [16, 17].

Periapical radiography, panoramic radiography, and cone‐beam computed tomography (CBCT) are the imaging techniques used to evaluate the presence of PALs [18]. CBCT provides a detailed three‐dimensional image of the area of interest, facilitating the visualization of roots and root canals and revealing fine anatomical structures, such as lateral canals and ramifications, as well as pulp stones, resorption defects, and root fractures [19]. CBCT has overcome the limitations of 2D imaging, such as distortion and superimposition of structures, and is less affected by the thickness of the cortical plate or trabecular bone density compared to 2D radiographs [20, 21]. Therefore, CBCT is the most accurate technique for detection of such lesions [14, 22]. CBCT evaluation of the periradicular tissues may help predict the prognosis and long‐term survival of the tooth [23]. In this context, it is also important to consider that CBCT allows for early diagnosis of endodontic failure, planning a reintervention, and evaluation of the presence of missed canals, which are crucial aspects in endodontic retreatment and treatment planning [24]. Furthermore, several published studies addressing similar or comparative topics highlight how CBCT investigations, even if retrospective, validate the strength of radiographic assessments in endodontics [25].

While numerous studies have investigated the relationship of PALs and various factors, including multirooted teeth, direct evaluation of each specific root in multirooted teeth has yet to be thoroughly addressed [26, 27]. Each root of a multirooted tooth may have a distinct outcome.

Considering the importance of understanding the relationship between the presence, volume, and size of a PAL with certain prognostic factors, and lack of precise evaluation of lesion size by using CBCT in previous studies, this cross‐sectional study was conducted aiming to investigate the association of factors such as missed canals, root filling length, perforation, broken instruments, restoration type (amalgam, composite, or prosthetic crown), and presence/absence of a cast/prefabricated post with the presence and size of PALs in mandibular anterior and premolar teeth. The information obtained in this study may serve as a guide for clinicians to identify the factors contributing to the development of PALs and understand the degree of influence of each factor independently. This will enable them to make more precise decisions in treatment planning and determination of prognosis, especially in retreatment cases.

## 2. Materials and Methods

The protocol of this cross‐sectional study adhered to the STROBE guidelines for cross‐sectional studies, and received approval from the ethics committee of Shahid Beheshti University of Medical Sciences (IR.SBMU.DRC.REC.1401.037).

### 2.1. Sample Size Calculation

The sample size was determined to be 105 teeth with/without PALs for estimation of the mean lesion volume, considering 95% confidence level, standard deviation of *σ* = 70 [28], and absolute error of estimation *d* = 15 units using the following formula where *Z* is the confidence coefficient from the normal distribution table (which is 1.96 for 95% confidence interval).

n=Z12−α/2×σ2d2.

### 2.2. Inclusion Criteria

The study included images of endodontically treated mandibular anterior and premolar teeth with or without PALs. The PALs had to be isolated with no apico‐marginal communication. All images had been captured using Soredex Scanora 3D CBCT scanner (Nahkelantie 160’ Tuusula, Finland) with 200 cm field of view, 200 μm voxel size, 6 mA tube current, and 90 kVp tube potential.

### 2.3. Exclusion Criteria

Images with poor quality, artifacts, endodontically untreated teeth, teeth with a history of previous endodontic surgery, teeth with lesions connecting to the roots of the adjacent teeth, teeth with strip perforations, teeth with dehiscence or fenestration, and teeth with root fractures were all excluded.

### 2.4. Study Process

Sixty‐three CBCT images of 105 mandibular anterior and premolar teeth with/without PALs that had undergone prior root canal treatment were evaluated. The images were selected from the archives of a specialized oral and maxillofacial radiology center in Tehran, Iran, from 2018 to 2022. The CBCT scans had been taken for endodontic purposes.

The images were analyzed using OnDemand software (Cybermed, Seoul, South Korea) version 2017 on a 14‐inch, 1920 × 1080 pixels resolution and 60 Hz monitor. The image contrast and brightness were optimized using the windowing tool.

A previously calibrated senior dental student assessed the endodontically treated teeth on CBCT images to identify relevant prognostic factors under the supervision of an experienced endodontist and an oral and maxillofacial radiologist. To assess the intraexaminer reliability, the examiner evaluated 30 samples for independent and dependent variables twice with a 10‐day interval, and the results were compared. If the examiner could not delineate the lesion borders, an experienced oral and maxillofacial radiologist reviewed the case and recorded the data.

**Figure 1:**
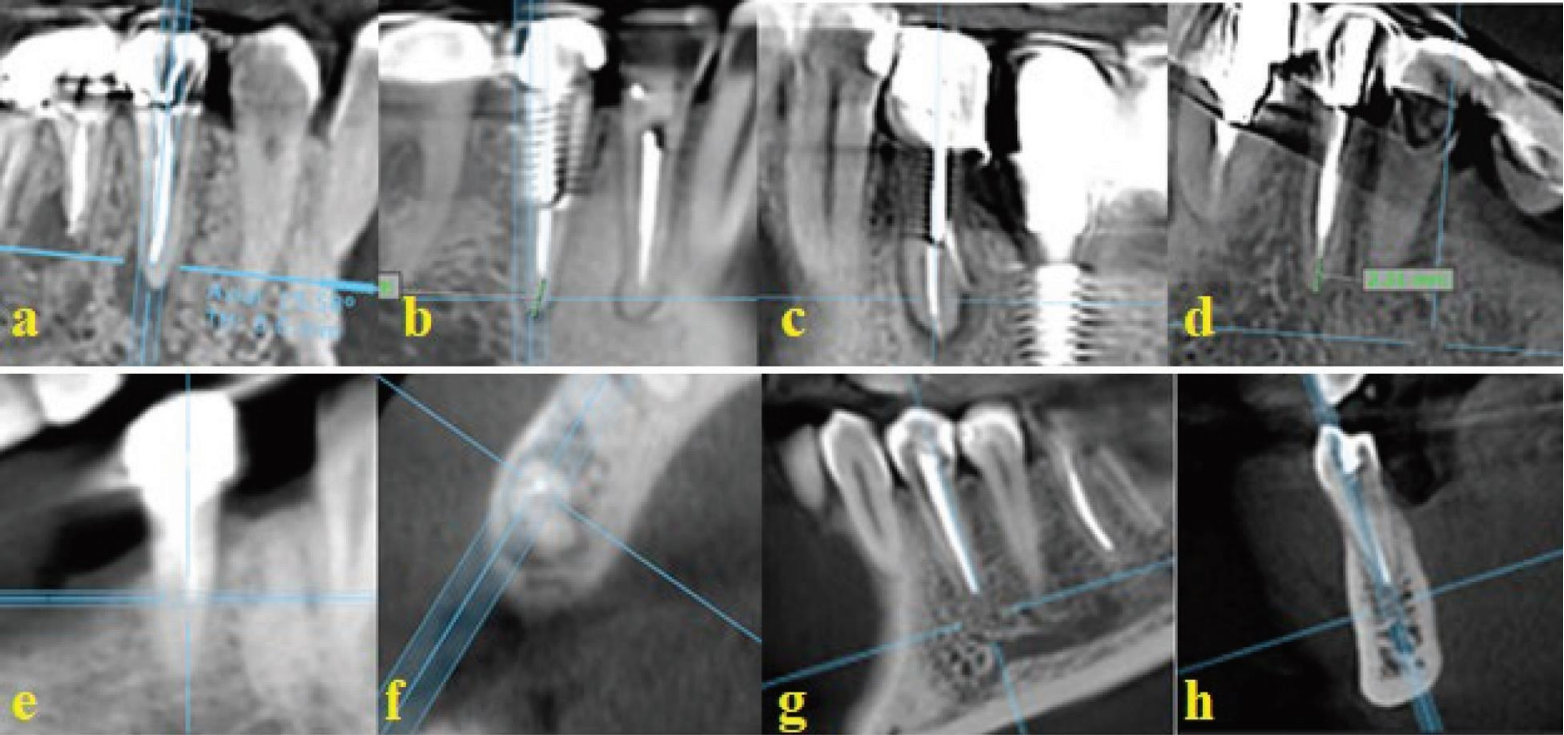
CBCT images demonstrating various endodontic procedural errors and anatomical variations: (a) coronal view of tooth #35 without any procedural error; (b) coronal view of tooth #35 with underfilling; (c) coronal view of tooth #35 with a perforation; (d) coronal view of tooth #35 with a broken instrument; (e) coronal view of tooth #45 with a missed lingual canal; (f) axial view of tooth #45 with a missed lingual canal; (g) coronal view of tooth #34 with overfilling; and (h) sagittal view of tooth #34 with overfilling.

The teeth of interest were examined for variables such as missed canals, perforation, broken instrument, root canal filling length and quality, presence/absence of cast/prefabricated post, and type of restoration (amalgam, composite, or prosthetic crown) on axial, coronal, and sagittal sections. The length of root filling was classified as appropriate if it ended within 0–2 mm from the apex, underfilled if shorter than 2 mm from the apex, and overfilled if the root filling material extruded and extended beyond the apex. Unfilled canal spaces were first examined on the axial view, and confirmed by evaluation of the sagittal and coronal views. Missed canals included any canal taking off from the main canal in the coronal, middle, or apical third, extending from the cementoenamel junction to the apex (Figure 1).

### 2.5. Data Collection

Data were collected regarding two dependent variables namely presence of PAL and its size (volume). A PAL was considered absent when the periapical bone structures were intact (diameter of periapical radiolucency < 0.5 mm). Otherwise, A PAL was considered to be present. The volume of PAL was measured manually using the area tool to determine the lesion cross‐sectional area on each cross‐sectional image, marked at 1 mm intervals. The total volume was calculated using the formula:

V=h×S12+√S12×S+S/33+h×S23+√S23×S+S/+…,

where *V* is the volume, *h* is the slice thickness, and S1, S2, and S3 are the lesion areas in consecutive slices.

### 2.6. Statistical Analysis

The collected data were presented using descriptive statistics, including tables, graphs, and measures of central tendency and dispersion. Multiple logistic regression was applied to examine the relationship between the independent and dichotomous dependent variables (presence/absence of PAL). The general linear model (GLM) with an identity link function was used to analyze the effect of independent variables (missed canal, overfilling, broken instrument, perforation, restoration type, and presence/absence of post) on the quantitative dependent variable (PAL volume).

The reliability of the measurement of qualitative variables (root filling length, presence of missed canals, perforation, broken instrument, presence of intracanal post, and coronal restoration type) was determined using the kappa (*κ*) coefficient, while the intraclass correlation coefficient (ICC) was used for quantitative variables and specifically PAL volume. A kappa value > 0.6 denoted acceptable reliability. An ICC > 0.75 indicated a reliable evaluator. The type I error was set at *α* = 0.05, and values below this threshold were considered statistically significant. Analyses were conducted using SPSS version 26.0 (IBM, Armonk, NY, USA).

## 3. Results

In this study, the ICC was 0.95 (*p*  < 0.001), demonstrating high reliability. The kappa coefficient was 0.896 for root canal filling length, 0.65 for broken instrument, and 1 for all other variables.

A total of 63 cases were examined, including 27 males and 36 females, with a mean age of 47.58 ± 10.25 years for females and 59.78 ± 14.28 years for males. Of all, 43 teeth (41%) had PALs (Tables 1 and 2).

**Table 1:** Descriptive information of the study population.

Independent variable	Category	Sample size, *n* (%)	PAL status		Mean PAL volume (mm^3^)
			Present, *n* (%)	Absent, *n* (%)	
Gender	Female	60 (57.1%)	26 (43.3%)	34 (56.7%)	6.39 (std = 10.98)
	Male	45 (42.8%)	17 (37.8%)	28 (62.2%)	9.932 (std = 21.04)
Missed canal	Present	3 (2.9%)	2 (66.6%)	1 (33.3%)	11.25 (std = 16.22)
	Absent	102 (97.1%)	41 (40.2%)	61 (59.8%	7.81 (std = 11.91)
Canal filling length	2–0.5 mm short of the apex	53 (50.5%)	11 (20.8%)	42 (79.2%)	6.8 (std = 15.9)
	More than 2 mm short of the apex	17 (16.2%)	10 (58.8%)	7 (41.2%)	6.77 (std = 11.87)
	Beyond the apex	23 (21.9%)	15 (65.2%)	8 (34.8%)	11.88 (std = 20.61)
Restoration type	Amalgam restoration	27 (25.7%)	12 (44.4%)	15 (55.6%)	8.41 (std = 14.24)
	Composite restoration	19 (18.1%)	6 (31.6%)	13 (68.4%)	4.67 (std = 10.62)
	Porcelain‐fused‐to‐metal crown	59 (56.2%)	25 (42.4%)	34 (57.6%)	8.71 (std = 18.27)
Broken instrument	Present	6 (5.7%)	3 (50%)	3 (50%)	18.03 (std = 26.37)
	Absent	99 (94.3%)	40 (40.4%)	59 (59.6%)	7.29 (std = 15.24)
Intracanal post	Absent	47 (44.8%)	18 (38.3%)	29 (61.7%)	6.71 (std = 12.77)
	Cast post in the canal	42 (40.0%)	20 (47.6%)	22 (52.4%)	9.62 (std = 18.88)
	Prefabricated post in the canal	16 (15.2%)	5 (31.3%)	11 (68.8%)	6.92 (std = 17.48)
Perforation	Present	10 (9.5%)	6 (60%)	4 (40%)	23.70 (std = 23.98)
	Absent	95 (90.5%)	37 (38.9%)	58 (61.1%)	6.24 (std = 14.21)

Abbreviation: PAL, periapical lesion.

**Table 2:** Prevalence and volume of PALs in different mandibular tooth types.

Tooth type	PAL		PAL volume + SD (mm^3^)
	Presence	Absence	
Mandibular central incisors	0 (0%)	2 (100%)	0
Mandibular lateral incisors	1 (25%)	3 (75%)	1.68 (SD = 3.36)
Mandibular canines	3 (21.5%)	11 (78.5%)	5.24 (SD = 10.45)
Mandibular first premolars	14 (43.8%)	18 (56.3%)	6.36 (SD = 13.39)
Mandibular second premolars	25 (47.2%)	28 (52.8%)	10.31 (SD = 19.18)

Abbreviations: PAL, periapical lesion; SD, standard deviation.

There was no significant correlation between age (*p* = 0.879), gender (*p* = 0.317), tooth type (*p* = 0.159), restoration type (*p* = 0.760), and presence of a cast/prefabricated post in the canal (*p* = 0.826) with the presence of PALs (Table 3).

**Table 3:** Effect of independent variables on presence of PALs using the multiple logistic regression test; female gender was chosen for comparison with the male gender, and premolar teeth were chosen for comparison with anterior teeth as the reference.

Independent variable	*B* ^a^	Std. error^b^	Wald	*p*‐Value	OR^c^	95% CI for OR	
						Lower	Upper
Gender	0.579	0.579	1.000	0.317	1.784	0.574	5.55
Age	−0.003	0.022	0.023	0.897	0.997	0.954	1.04
Presence of missed canal	3.419	1.684	4.122	0.042	30.541	1.26	828.452
Canal filling length	—	—	17.753	0.000	—	—	—
Canal filling length (underfilled)	2.544	0.740	11.816	0.000	12.729	2.984	54.292
Canal filling length (overfilled)	3.215	0.793	16.440	0.000	24.897	5.263	117.772
Perforation	2.152	0.925	5.419	0.020	8.606	1.405	52.703
Presence of cast/prefabricated post in the canal	—	—	0.382	0.826	—	—	—
Restoration	—	—	0.055	0.760	—	—	—
Broken instrument	2.152	1.104	4.535	0.033	10.497	1.405	91.370
Tooth type	1.150	0.817	1.982	0.159	3.158	0.637	15.65

^a^Coefficient value: coefficient of the independent variable in the model.

^b^Standard error of the coefficient: this measure assesses the precision of the estimated coefficient.

^c^Odds ratio (OR): this term refers to the measure of association between the dependent and independent variables.

Factors like missed canals (*p* = 0.042), underfilled canals (*p*  < 0.001), overfilled canals (*p*  < 0.001), perforation (*p* = 0.020), and broken instrument (*p* = 0.033) significantly affected the presence of PAL. The odds ratio (OR) for missed canals was 30.541, indicating a 30 times higher likelihood of PALs in case of presence of missed canals. Overfilled canals had an OR of 24.897, and underfilled canals had an OR of 12.729. Broken instruments (OR = 10.497) and perforation (OR = 8.606) also increased the likelihood of PALs (Table 3).

In the GLM analysis, unfilled canals (*p* = 0.311), underfilled canals (*p* = 0.450), presence of a prefabricated post (*p* = 0.457), intracanal cast post (*p* = 0.926), and restoration type (*p* = 0.734 for prosthetic crown, *p* = 0.735 for composite restoration, and *p* = 0.527 for amalgam restoration) did not show a significant relationship with PAL volume. However, gender (*p* = 0.047), overfilling (*p* = 0.013), broken instrument (*p*  < 0.001), and perforation (*p* = 0.009) were positively correlated with larger PALs (Table 4). The PAL volume was about two times larger in case of overfilling (mean = 11.88) and three to four times larger in presence of broken instrument (mean = 18.03) and perforation (mean = 23.7) compared to absence of these errors.

**Table 4:** Effect of independent variables on PAL volume in the GLM analysis.

Independent variable	*B* ^a^	Std. error^b^	Wald	*p*‐Value
Perforation	21.747	4.9786	19.080	0.000
Broken instrument	17.175	6.5319	6.914	0.009
Overfilling	9.273	3.7205	6.212	0.013
Gender (male)	6.723	3.3897	3.934	0.047
Age	−0.229	0.1268	3.249	0.071
Missed canal	8.957	8.8348	1.028	0.311
Crown restoration	1.577	4.6469	0.115	0.734
Composite restoration	−1.532	4.5316	0.114	0.735
Underfilling	2.714	3.5948	0.570	0.450
Prefabricated post	−3.285	4.4147	0.554	0.457
Cast post	−0.416	4.4784	0.009	0.926
Tooth type	−0.283	4.0533	0.005	0.944

*Note*: In this model, amalgam restoration, female gender, and anterior teeth were used as the reference for the purpose of comparison.

^a^Coefficient value: coefficient of the predictor variable.

^b^Standard error of the coefficient: this measure assesses the precision of estimated coefficient.

## 4. Discussion

In contemporary endodontics, CBCT images have significantly enhanced evaluation of dental anatomy, quality of root canal treatment, and detection of procedural errors [29]. This study conducted a thorough evaluation of the association between specific prognostic factors, including procedural errors such as missed canals, apical perforation, variations in obturation length, presence of intracanal posts or screws, restoration type, and fractured instruments, and the periapical status of endodontically treated mandibular anterior and premolar teeth. Given that both the presence and extent of PALs are critical determinants of endodontic treatment outcomes, these factors hold substantial relevance in guiding appropriate treatment planning and prognostic assessment. Thus, detection of PALs, accurate determination of their size, and finding the influential factors in their development may enable more accurate treatment planning and estimation of treatment prognosis.

Epidemiological studies have reported different prevalence rates for PALs in endodontically treated teeth. The prevalence of PALs was found to be 40.9% in the present study, which was close to the prevalence rates reported in previous studies conducted in Belarus [30], Belgium [31], Brazil [32], Canada [33], Denmark [34], Japan [35], Lithuania [36], Scotland [37], Turkey [38], and the United States [39]. According to a recent systematic review and meta‐analysis by Tibúrcio‐Machado et al. [40], the global prevalence of AP is approximately 52% at the individual level and 39% in endodontically‐treated teeth, highlighting that AP remains a highly prevalent condition worldwide. Differences in prevalence rates observed among studies, such as those conducted in Germany [41] and Greece [42], may be attributed to geographical, methodological, and socioeconomic factors. Conducting studies with standardized methodologies across diverse populations may improve the comparability and reliability of prevalence data.

The present study found no significant correlation between age and presence of PALs. Images of individuals between 29 and 86 years were evaluated in this study, making it challenging to obtain precise results regarding the relationship of age and presence of PALs, especially since the number of individuals from different age groups was not the same. Similarly, Tibúrcio‐Machado et al. [40] reported that the relationship between age and AP was highly variable across studies and usually did not affect the prevalence of PAL.

Regarding gender, no significant correlation was found between gender and presence of PALs, consistent with the results of studies by Ng et al. [12], Georgopoulou et al. [42], Kirkevang et al. [43], and Boucher et al. [44]. Conversely, Jakovljevic et al. [6] found that male patients were more susceptible to development of PALs than females, probably due to better adherence of females to oral hygiene measures and their higher frequency of dental visits.

Tooth type (anterior/premolar) did not significantly correlate with the prevalence of PALs in the present study, similar to study by Jiménez‐Pinzón et al. [45]. They evaluated fewer anterior teeth (*n* = 20) compared to endodontically treated premolars (*n* = 85), but the prevalence of PALs was not significantly different between the two groups.

In the current study, the prevalence of missed canals was 2.8%; out of which, 66.6% were associated with PALs. This finding contrasts with the results of Baruwa et al. [16], who reported a prevalence of 5% for PALs in anterior and mandibular premolars due to a much larger sample size in their study (over 2000 samples). The small sample size in the present study calls for further research to accurately determine the prevalence of missed canals and PALs.

Comparison of over‐filling, under‐filling, and normally obturated teeth (within 0–2 mm distance from the apex) revealed that PALs were more common in over‐filled and under‐filled teeth. Moura et al. [46] found that obturations extending about 1 mm beyond the apex on CBCT scans had the worst prognosis, with a 16‐fold increase in the prevalence of PALs. Teeth with a root filling length more than 2 mm short from the radiographic apex (underfilled) ranked next in terms of having the worst prognosis, consistent with the present results.

Furthermore, the present study highlighted the potential consequences of overfilled root canals, indicating the increased risk of tissue damage due to over‐instrumentation and generation of inflammatory responses as the result of extrusion of filling materials or residual infection in the apical region. Root canal filling materials can also induce a foreign body reaction, leading to apical lesions even without bacterial involvement, thereby compromising the treatment prognosis [47].

Conversely, under‐filled root canals may result from the inability to debride the apical third of the canal (canal obstruction) or accumulation of infected dentinal chips, compromising the treatment prognosis due to the likelihood of persistent infection [48].

Procedural errors, and particularly presence of broken instruments in the canal, significantly increase the occurrence of PALs by compromising proper cleaning and obturation of the canal [49]. The current study also found a significant correlation between broken instruments and PALs in the linear regression analysis. However, the results regarding broken instruments have been inconsistent throughout the literature. For instance, McGuigan et al. [50] reported that broken instruments did not significantly affect endodontic treatment success in case of absence of a pre‐existing PAL but significantly decreased the success rate if a PAL was present before the treatment. Simon et al. [51] emphasized that the impact of broken instruments on treatment prognosis depends on the degree of canal disinfection before the incident, and the severity of pre‐existing infection. It is also worth noting that detection of broken files on CBCT scans, especially in endodontically treated teeth, is challenging due to the artifacts caused by the high‐density root canal filling materials.

The present study also found a significant increase in the occurrence of PALs associated with apical perforations, with an almost 8.5‐fold higher likelihood. This finding is consistent with previous research. Krupp et al. [52] noted that although sealing of the perforated area with mineral trioxide aggregate affects the treatment success, there remains a 30% risk of failure and PAL formation, indicating a reduced overall quality of treatment in cases with perforations. Lin et al. [53] emphasized that treatment failures associated with perforations primarily occur due to the inability of instruments to reach the area apical to the perforation site. Farzaneh et al. [54] found significantly different treatment outcomes (absence of lesions, absence of symptoms) in teeth with and without AP (86% and 36%, respectively), with much poorer treatment prognosis in teeth with preoperative perforations.

Out of the 58 teeth with intracanal posts or screws, 25 exhibited PALs, and no significant association was observed between the presence of a cast/prefabricated post and prevalence of PALs. While some studies reported a significant correlation between the use of posts in restored teeth and an increased prevalence of AP, others found no such association [55–57]. In contrast to the current findings, Boucher et al. [44] reported that 28% of teeth with posts had PALs, which was only marginally higher than the 23% rate observed in the current study. This discrepancy in the results may stem from the differences in the methods used to evaluate the presence of PALs. Boucher et al. [44] used periapical radiographs, which may have misclassified some teeth without posts as lesion‐free due to the lower accuracy of this imaging technique, potentially influencing the statistical significance of the results.

Based on the present findings, it appears reasonable to conclude that presence of an intracanal post alone does not significantly impact the success of root canal treatment. Other factors, such as proper root canal cleaning, pretreatment presence of a PAL, and quality of root canal obturation may play a more critical role in determining the treatment outcome than the mere presence of an intracanal post [58, 59].

In the present study, the regression analysis did not show any correlation between the restoration type and presence of PALs. This finding was consistent with the results of Hommez et al. [60], who reported that restoration type had no significant effect on the periapical status of the teeth with optimal obturation. Similarly, Gambarini et al. [61] reported that the quality of obturation had a more significant influence on the long‐term success of endodontic treatment than the restoration type. Achieving a good coronal seal and preventing recurrent caries are the primary factors affecting the periapical health of endodontically treated teeth, and not the restorative material used. Additionally, if the obturation quality is poor, a coronal seal alone cannot ensure the periapical tissue health [5]. However, Meirinhos et al. [7] found that PALs had a significantly lower frequency in teeth restored with intracoronal restorations compared to those restored with full crowns. They attributed this difference to the greater coronal destruction in teeth requiring full crowns prior to endodontic treatment.

Considering the small number of endodontically treated anterior teeth in the present study, along with the low incidence of PALs in this group and the findings of other studies that primarily attributed the endodontic treatment success to the obturation quality rather than the restoration type [62], lack of a significant association between the restoration type and PALs in the present study appears logical.

Various studies in the literature have measured the volume of 3D objects, including PALs. Kriesler et al. [63] evaluated lesion volume by measuring the saline volume required to fill the bone defect during surgery. Von Arx et al. [64] calculated the volume of lesions postoperatively by multiplying the bone defects’ mesiodistal, apico‐coronal, and buccolingual dimensions. Only Bieszczad et al. [65] assessed lesion volume using CBCT scans. In the current study, the lesion volume was measured by sectioning the lesion at 1 mm intervals and summing the volumes of all sections, providing a higher accuracy compared to previous studies like the one conducted by Hajihassani et al. [25], where the largest lesion diameter was measured in three dimensions (buccolingual, mesiodistal, and occluso‐apical) and compared. In the current study, the mean lesion volume was 7.90 mm^3^.

No significant correlation was found between age and PAL volume in the current study, which was in contrast to the results of Hajihassani et al. [25]. The reason can be the type of study samples, different age range of patients, and lack of studies on the relationship of age and PAL volume. In general, bone resorption increases and bone density decreases with age, resulting in an increase in bone destruction by root inflammatory lesions [66].

However, a significant relationship was observed between gender and PAL volume, consistent with the results of Hajihassani et al. [25], and Kazemipoor et al. [66]. In the present study, PAL volume was larger in males (9.932 mm^3^) than females (6.39 mm^3^), possibly due to females’ greater attention to oral health and earlier detection of PALs in their routine dental imaging. Thus, the observed lesions were smaller in females. The largest mean PAL volume was associated with perforated teeth (23.70 mm^3^), indicating a significant relationship between perforation and PAL volume. PAL volume ranked the second largest in teeth with broken instruments (18.03 mm^3^), showing a significant correlation between this procedural error and PAL volume (*p* = 0.009). However, further investigations are required due to the cross‐sectional design of the present study, lack of knowledge about the pretreatment status and timing of instrument fracture, and absence of previous studies on the correlation of PAL volume and broken instrument.

The GLM analysis showed a significant correlation between over‐filling and PAL volume (*p* = 0.013), with the highest mean PAL volume observed in roots with perforations (23.70 mm^3^) followed by broken instruments (18.03 mm^3^) and over‐filled roots (11.88 mm^3^). In the present study, the likelihood of a PAL was higher in over‐filled compared to under‐filled teeth, with larger lesions and a potentially poorer prognosis in over‐filled teeth. Moura et al. [46] showed the worst treatment prognosis in teeth where root fillings extended 1 mm beyond the apex. This finding may be due to the extruded root canal filling materials causing foreign body reactions in the periapical area, leading to development of new lesions and disrupting the healing process of the existing lesions, resulting in larger lesions [67].

The present results also showed that the mean lesion volume in under‐filled roots (6.77 mm^3^) was not significantly different from that in inappropriately filled teeth. Generally, root fillings shorter than 2 mm from the root apex result in inadequate debridement and 3D sealing of the apical region, promoting the growth and proliferation of microorganisms and development of PALs [7]. Considering the cross‐sectional design of this study, and lack of information about the presence/absence or size of PALs prior to the treatment onset, further investigations are required on this topic.

No significant correlation was found between missed canals and PAL volume in the present study (*p* = 0.311), possibly due to the low prevalence of missed canals in this study. In terms of PAL volume, teeth with missed canals (11.25 mm^3^) ranked fourth after perforations, broken instruments, and over‐filling. PAL volume and growth rate are expected to be higher in teeth with active infection, or untreated, or inadequately treated canals [68].

There was no significant correlation between the presence of intracanal cast posts (*p* = 0.926), presence of prefabricated posts (*p* = 0.457), restoration type (*p* = 0.734), or tooth type (*p* = 0.944) with PAL volume. Overall, since this study was the first to examine the relationship between these variables and PAL volume, no other studies were found for the purpose of comparison.

This study highlighted the factors affecting the root canal treatment outcome, emphasizing on the importance of technical details and the impact of missed canals, root filling extension, and apical perforations on the occurrence of PALs. These findings enhance the understanding of root canal treatment complexities and underscore the importance of refined techniques to maximize treatment success.

## 5. Limitations, Strengths, and Recommendations

The cross‐sectional design of this study posed significant limitations. It was impossible to determine whether the observed lesion on the CBCT scan developed after endodontic treatment, was progressing, or healing. This limitation stems from the unknown status of the periapical status and pulp condition before the treatment; it was not known whether the pulp was vital or necrotic. Also, patient history with respect to pathological conditions and systemic diseases before endodontic treatment was unknown. Moreover, information about other confounders that can affect the treatment outcome such as clinical symptoms, that is, preoperative pain, medication intake by patients before and after endodontic treatment, method of obturation (lateral compaction or warm vertical condensation), and the operator’s experience and skills was lacking. This knowledge gap might have affected the results. The timing of radiography relative to the endodontic treatment time was also unclear. Specifically, the interval between the endodontic treatment and CBCT imaging was unknown, adding another layer of uncertainty to the study conclusion. Finally, this cross‐sectional study was conducted on the CBCT scans available in the archives; thus, caution is advised when generalizing the present results to the general population of Tehran. Ideally, the study should have been conducted in a random setting. However, this was not possible due to the fact that CBCT imaging of healthy individuals only for research purposes is not ethical according to the European Society of Endodontology guideline. Despite these limitations, this study had several strengths. It enabled detailed evaluation of various prognostic factors such as missed canals, perforations, obturation length, presence of cast/prefabricated post in the canal, restoration type, and broken instrument in endodontically treated teeth. This comprehensive analysis allowed for thorough understanding of the factors influencing the endodontic treatment outcome. The study also focused on the correlation between procedural errors and periapical status, which is crucial for monitoring the root canal treatment outcome. By identifying these correlations, the study highlighted the importance of addressing procedural errors to maximize treatment success. Inclusion of a wide age range (29–86 years) allowed for an inclusive assessment of different age groups, even though the distribution was not equal. This broad age range provided insights into how endodontic treatment outcomes might affect various age groups. To build upon these findings, future studies should employ longitudinal designs, such as case‐control or cohort studies, focusing on the pretreatment status of the pulp and periapical tissues, clinical symptoms, systemic conditions, and the timing of treatment initiation. Additionally, using the existing sample for a complementary study where all canals are retreated by one single calibrated operator and monitoring the regression of PALs over time would provide valuable insights. Such monitoring should also correlate lesion regression with specific iatrogenic factors, including missed canals, instrument fracture, and inadequate foraminal disinfection to generate more robust and clinically meaningful data. Based on the results of this study, similar investigations using routinely collected periapical images in clinical practice are also recommended. These would provide practical insights and allow for a more accurate assessment of factors influencing PAL development, ultimately guiding clinicians in treatment planning and prognostic evaluation.

## 6. Conclusions

This study highlighted the important role of CBCT in modern endodontics, especially for evaluation of dental anatomy, assessing the quality of root canal treatment, and identifying procedural errors. Comprehensive evaluation of prognostic factors revealed no significant correlation between age, gender, or tooth type with the prevalence of PALs. However, presence of missed canals, overextended root fillings, and procedural errors such as broken instrument and apical perforation significantly affected the periapical status.

Regarding the lesion volume, no significant correlation was found with age, tooth type, missed canals, restoration type, or presence of an intracanal post or screw. However, apical perforation, broken instrument, overextended fillings, and gender had a significant association with PAL volume.

## Author Contributions

Nazanin Zargar and Ayeh Etemadi contributed to the selection of the research topic and was responsible for drafting and writing the manuscript. Ali Akbar Ahmadi and Yaser Safi performed data collection. Alireza Akbarzadeh Baghban participated in data analysis and interpretation. Seyedeh Sareh Hendi supervised the study and provided critical revisions to the manuscript.

## Funding

This work was supported by the Research Centre of Shahid Beheshti University of Medical Sciences (Grant 4090).

## Disclosure

All the authors have reviewed and approved the final version of the manuscript. This funding has been devoted just to purchase materials used in their study.

## Ethics Statement

The received approval from the ethics committee of the Shahid Beheshti University of Medical Sciences (IR.SBMU.DRC.REC.1401.037).

## Conflicts of Interest

The authors declare no conflicts of interest.

## Data Availability

The data that support the findings of this study are available from the corresponding author upon reasonable request.
